# Association of cerebral malaria and TNF-α levels: a systematic review

**DOI:** 10.1186/s12879-020-05107-2

**Published:** 2020-06-23

**Authors:** Luana Leão, Bruna Puty, Maria Fâni Dolabela, Marinete Marins Povoa, Yago Gecy De Sousa Né, Luciana Guimarães Eiró, Nathália Carolina Fernandes Fagundes, Lucianne Cople Maia, Rafael Rodrigues Lima

**Affiliations:** 1 Laboratory of Functional and Structural Biology, Institute of Biological Sciences, Federal University of Pará, 01 Augusto Correa Street, Guama, Belem, PA 66075-900 Brazil; 2 Postgraduate Program in Pharmaceutical Sciences, Institute of Health Sciences, Federal University of Pará, Belém, Brazil; 3 Section of Parasitology, Institute of Evandro Chagas, Levilândia, Brazil; 4 School of Dentistry, Faculty of Medicine and Dentistry, University of Alberta, Edmonton, Canada; 5 Department of Pediatric Dentistry and Orthodontics, School of Dentistry, Federal University of Rio de Janeiro, Rio de Janeiro, Brazil

**Keywords:** *P. falciparum*, Malaria, TNF-α, Cerebral malaria, Systematic review

## Abstract

**Background:**

Cerebral malaria is the most severe form of infection with *Plasmodium falciparum* characterized by a highly inflammatory response. This systematic review aimed to investigate the association between TNF-α levels and cerebral malaria.

**Methods:**

This review followed the Preferred Reporting of Systematic Review and Meta-analyses (PRISMA) guidelines. The search was performed at PubMed, LILACS, Scopus, Web of Science, The Cochrane Library, OpenGrey and Google Scholar. We have included studies of *P. falciparum*-infected humans with or without cerebral malaria and TNF-α dosage level. All studies were evaluated using a risk of bias tool and the GRADE approach.

**Results:**

Our results have identified 2338 studies, and 8 articles were eligible according to this systematic review inclusion criteria. Among the eight articles, five have evaluated TNF- α plasma dosage, while two have evaluated at the blood and one at the brain (post-Morten). Among them, only five studies showed higher TNF-α levels in the cerebral malaria group compared to the severe malaria group. Methodological problems were identified regarding sample size, randomization and blindness, but no risk of bias was detected.

**Conclusion:**

Although the results suggested that that TNF-α level is associated with cerebral malaria, the evidence is inconsistent and imprecise. More observational studies evaluating the average TNF-alpha are needed.

## Background

Malaria is an acute infection caused by the protozoan parasite *Plasmodium*. Over 100 species of *Plasmodium* can infect several groups of vertebrates like reptiles, birds, and mammals. However, only six of them are capable of infecting humans: *Plasmodium vivax*, *Plasmodium ov*er, *Plasmodium malariae*, *Plasmodium knowolesi*, *Plasmodium simiu* and *Plasmodium falciparum* [1–6].

Among these species, the *P. ovale* is the least common. It is primarily concentrated in Western Africa, while *P. malariae* is widely distributed with low incidence. Infection by *P. vivax* and *P. falciparum* is the most common and can cause severe anemia. However, only *P. falciparum* may lead to the most severe form of the disease, cerebral malaria (CM) [3, 7–9]. The CM occurs in about 1% of registered cases of the disease, with 2 million deaths worldwide [10].

The main obstacle to eradicating malaria are the decrease of international and domestic funding, the presence of endemic malaria regions, anomalous weather patterns, parasite resistance of treatment, and mosquito resistance of insecticides [11]. Countries that have had succeeded or are in progress of malaria control relate success with early diagnosis and rapid and effective treatment [11].

In 2016, 216 million cases of malaria were registered in 91 countries, with 445.000 deaths. Among the cases, 80% came from countries are in sub-Saharian Africa [11]. The first clinical symptoms of malaria are nonspecifically starting with the breakage of parasitized erythrocytes that release antigens into the blood, giving start to the host’s immunologic response. Children with severe malaria frequently evolved to one or more symptoms, as are the following: severe anemia, respiratory deficit related to metabolic acidosis or cerebral malaria. In contrast, adults often have organs severely compromised [11, 12].

According to the World Health Organization [11], CM is an encephalopathy with a coma associated with neurologic disorders due to hemorrhage [13]. Patients who have survived to CM show permanent neurologic complications as cognitive and speech deficits, motor alterations and cortical blindness [14, 15]. It is essential to note that the development of CM remains unclear, with only two hypotheses that seek to clarify it [1, 10, 16–20].

A study in 2006 proposed the unification of theories, suggesting that cerebral malaria is a consequence of hypoxia since parasitized erythrocytes adhere to the endothelium wall and induce the blockage of blood flow [1, 7, 21]. On the other hand, the inflammation theory suggests CM is induced by a high inflammation response initiated by monocytes activation and the induction of pro-inflammatory mediators as interleukins (IL), macrophage colony-stimulating factor (M-CSF), tumour necrosis factor-alpha (TNF-α) and lymphotoxin [22].

Among them, the TNF-α secretion by macrophages and T CD4^+^ has gotten attention. TNF-α production on the initial CM phase seems to be related to a reduction of parasitic load. However, the overproduction of TNF-α in the late phase is associated with disease severity [23]. This dual role of TNF-α suggests that the regulation and time production of pro-inflammatory cytokines are essential to infection control [23]. On endemic malaria areas, children under 5 years old are more susceptible to infection and death, responsible for more than two thirds (70%) of all malaria deaths in this age group [24].

In this way, this systematic review aimed to gather clinical evidence of the association of cerebral malaria by *Plasmodium falciparum* and TNF-α level increasing in humans.

## Methods

### Protocol and registration

This systematic review was registered on the International prospective register of systematic reviews (PROSPERO), under the number CRD42016042745. The *Preferred Reporting of Systematic Review and Meta-analyses* (PRISMA) were followed [25].

### Strategy search, selection and eligibility criterion

From July to September 2018, searches were performed on the following literature bases: PubMed, Latin American and Caribbean Health Sciences Literature database (LILACS), Scopus, Web of Science, The Cochrane Library, OpenGrey and Google Scholar. There was no restriction regarding language or year of publication. In each database, a specific search strategy, combining MESH and free terms was devolved following the rules of each database (Table 1). After the inclusion of selected studies, we performed a hand search on each study’s reference list. Also, alerts were created on each database.

**Table 1:** Mesh and terms used on searching for articles in the databases

Data base	Search strategy	
**#1 AND #2**		
**PubMed**	**#1** Search (((((((((((((((((Tumor Necrosis Factor-alpha [MeSH Terms]) OR Tumor Necrosis Factor-alpha [Title/Abstract]) OR TNF-α [Title/Abstract]) OR Tumor Necrosis Factor alpha [Title/Abstract]) OR TNF Superfamily, Member 2[Title/Abstract]) OR Cachectin-Tumor Necrosis Factor [Title/Abstract]) OR Cachectin Tumor Necrosis Factor [Title/Abstract]) OR TNFalpha [Title/Abstract]) OR TNF-alpha [Title/Abstract]) OR Tumor Necrosis Factor [Title/Abstract]) OR Tumor Necrosis Factor Ligand Superfamily Member 2[Title/Abstract]) OR Cachectin [Title/Abstract]) OR Tumor Necrosis Factors [MeSH Terms]) OR Tumor Necrosis Factor Superfamily Ligands [Title/Abstract]) OR Necrosis Factors, Tumor [Title/Abstract]) OR TNF Receptor Ligands [Title/Abstract]) OR Receptor Ligands, TNF [Title/Abstract]) OR Tumor Necrosis Factors [Title/Abstract]	**#2** ((((((Humans [MeSH Terms]) OR Humans [Title/Abstract]) OR Man (Taxonomy)[Title/Abstract]) OR Man, Modern [Title/Abstract]) OR Modern Man [Title/Abstract]) OR *Homo sapiens* [Title/Abstract]) OR Human [Title/Abstract]
**#1 AND #2**		
**Scopus**	#1 TITLE-ABS-KEY (“Tumor Necrosis Factor-alpha”) OR TITLE-ABS-KEY (tnf-α) OR TITLE-ABS-KEY (“Cachectin Tumor Necrosis Factor”) OR TITLE-ABS-KEY (tnfalpha) OR TITLE-ABS-KEY (“Tumor Necrosis Factor”) OR TITLE-ABS-KEY (“Tumor Necrosis Factor Ligand Superfamily Member 2”) OR TITLE-ABS-KEY (cachectin) OR TITLE-ABS-KEY (“TNF Receptor Ligands”)	**#2** TITLE-ABS-KEY (humans) OR TITLE-ABS-KEY (“Modern Man”) OR TITLE-ABS-KEY (“Homo sapiens”) AND TITLE-ABS-KEY (“Cerebral Malaria”) OR TITLE-ABS-KEY (“Malaria Meningitis”) OR TITLE-ABS-KEY (“*Plasmodium falciparum*”) OR TITLE-ABS-KEY (“Malaria, Falciparum”) OR TITLE-ABS-KEY (“Plasmodium falciparum Malaria”)
**#1 AND #2**		
**Cochrane**	**#1** Tumor Necrosis Factor-alpha OR tnf-α OR Cachectin Tumor Necrosis Factor OR tnfalpha OR Tumor Necrosis Factor OR Tumor Necrosis Factor Ligand Superfamily Member 2	***#2*** humans OR Modern Man OR Homo sapiens AND Cerebral Malaria OR Malaria Meningitis OR Plasmodium falciparum OR Malaria, Falciparum OR Plasmodium falciparum Malaria in Title Abstract Keyword - (Word variations have been searche
**#1 AND #2**		
**Web of Science**	#1 Tópico: (Tumor Necrosis Factor-alpha) OR Tópico: (TNF-α) OR Tópico: (Cachectin Tumor Necrosis Factor) OR Tópico: (TNFalpha) OR Tópico: (Tumor Necrosis Factor*) OR Tópico: (Tumor Necrosis Factor Ligand Superfamily Member 2) OR Tópico: (Cachectin) OR Tópico: (TNF Superfamily, Member 2) OR Tópico: (TNF Receptor Ligands) OR Tópico: (Tumor Necrosis Factor Superfamily Ligands)	**#2** Tópico: (Human*) OR Tópico: (Modern Man) OR Tópico: (Man (Taxonomy)) OR Tópico: (Homo sapiens)
**Open Grey**	TNF AND HUMAN AND CEREBRAL MALARIA	
**#1 AND #2**		
**LILACS**	#1 (tw:(Tumor Necrosis Factor-alpha)) OR (tw:(TNF-α)) OR (tw:(Cachectin Tumor Necrosis Factor)) OR (tw:(TNFalpha)) OR (tw:(Tumor Necrosis Factor$)) OR (tw:(Tumor Necrosis Factor Ligand Superfamily Member 2)) OR (tw:(Cachectin)) OR (tw:(TNF Receptor Ligands))	#2 (tw:(Human$)) OR (tw:(Modern Man)) OR (tw:(Homo sapiens)) AND (tw:(Cerebral Malaria)) OR (tw:(Malaria Meningitis)) OR (tw:(Plasmodium falciparum$)) OR (tw:(Malaria, Falciparum)) OR (tw:(Plasmodium falciparum Malaria))
**Google Scholar**	“Tumor Necrosis Factor-alpha” AND TNF-α AND “Cerebral Malaria” AND HUMANS AND NOT BOOKS AND NOT ANIMAL AND NOT “IN VITRO” AND NOT REVIEW AND NOT SYSTEMATIC REVIEW	

After searches, duplicated studies were identified and excluded in a reference manager software (EndNote X7, Thomson Reuters). The selection of studies was performed independently by two reviewers (LKRL and BP) according to the following PECO: (**P- p**opulation) Humans; (**E- e**xposition) presence of cerebral malaria induced by *Plasmodium falciparum*; (**C- c**omparison) presence of severe malaria but no cerebral commitment; (**O- o**utcome) Association between TNF-α immunologic response on a blood sample from patients that have been infected by *Plasmodium falciparum* and have developed cerebral malaria.

We first selected studies by title and abstract and then by full reading. A third reviewer (RRL) was consulted in case of disagreement. To the final selection of eligible studies, we took into consideration studies that have infected groups (by *P. falciparum* and CM) and reference group (infected by *P. falciparum* with no CM). We also included studies with TNF-α level dosage, at least in one sample. We excluded reviews, animals, and in vitro studies, clinical cases, case series, guidelines and editorials.

### Data extraction and risk of bias

After the selection of studies by full reading, we performed data extraction taking into account the region, infected population by *P. falciparum* with or without the development of CM, criterion for group characterization, collection methods and data analysis.

The methodological quality and risk of bias followed the protocol by Fowkes and Fulton’s [26] of a medical study. This protocol is based on questions about experimental design as a sample, presence of reference group (infected by *P. falciparum,* without CM), quality of methodology and results, withdrawals or loss of sample, experimental bias and confounding factors. Each question was answered according to the following code: (0) no problem; (+) minor problem; (++) major problem; (NA) not applicable (Table 2).

**Table 2:** Domains and risk of bias considered in risk of bias evaluation, according to Fowkes and Fulton [26]

Guideline	Checklist	Description
Is the study design appropriate to objectives?	objective common design	The type of study was marked in the appropriate \type of study. If the type of study was appropriate according to the study design was marked as “0” and as “++” if it was not appropriate.
	prevalence of Cross-sectional	
	Prognosis Cohort	
	Treatment Controlled trial	
	Cohort, case-control, cross-sectional	
Study sample representative?	Source of sample	It was identified as (0) when the origin was described in detail, a minor problem (+) when the study inaccurately cites the studied population region and a larger problem (++) was identified when the origin of the population was not mentioned sample.
	Sampling method	The item was assigned (0) for a full description of the sampling method, (+) for poor or no description of sample method, with no problem in matching between groups and (++) for poor or no description of sample method, interfering in the matching of the groups.
	Sample size	When the sample calculation was representative of the group, it was identified as (0). It was identified as a minor problem (+) when the sample calculation was not representative of the population under analysis, or when the study did not use a sample calculation. A larger problem (++) was identified when there was no sample calculation, and when the sample was < 50.
	Entry criteria/exclusion	The presence or absence of the points determined as important for eligibility in this review was assessed. The diagnosis of *P. falciparum* malaria, with groups with cerebral malaria and without cerebral malaria, was taken into account. Both sexes of all age groups were included, except pregnant women and participants identified with comorbidities and co-infected. For this criterion, it was identified as a minor problem (+) when two exclusion factors (pregnant women, comorbidities and co-infection) and a major problem (++) were present in the control group or the study group when two or more factors are present.
	Non-respondents	(0) was attributed to the studies that presented up to 30% positive responses by the population to participate in the studies, a minor problem (+) when there was a refusal, not being able to compromise the sample and a bigger problem (++) when there were refusal and commitment in sampling; in cross-sectional studies was assigned (NA)
Is the Control group acceptable?	Definition of controls	It was considered (0) studies that +showed control infected with P. falciparum, without cerebral malaria, a minor problem (++) when it was not clear the severity of the disease in the control group and a problem.
	Source of controls	It was identified as (0) when the origin was described in detail, a minor problem (+) when the study inaccurately cites the studied population region and a larger problem (++) was identified when the origin of the population was not mentioned sample.
	Matching/randomization	Studies with paired groups were considered a minor problem (+) in situations where it was not clear how the samples were paired, but the groups were paired with a larger problem (++) when there was no description or matching of sample groups.
	Comparable characteristics	We considered (0) studies that had sample groups with comparable characteristics and (++) when the study variables could not be matched and / or comparable.
Quality of measurements and outcomes?	Validity	(0) when the methods applied were appropriate for the study; was assigned a minor (+) problem when only one method for the analysis was used, but with reliable characteristics for the desired objective and a larger problem (++) when the methods used were not sufficient to meet the objective of the study.
	Reproducibility	(0) was identified when the evaluation of the methods was clearly described. They were considered as problems when the malaria diagnostic evaluation methods were not well described, do not describe how the sample was considered regarding the severity level of the disease, do not tell the type of infecting parasite, the lack of information regarding the quantification tests of TNF-α. A minor problem (+) when one of the previous items were present. A larger problem (++) when in presence two or more of the previous factors present.
	Blindness	It was considered (0) when the study presented blinding and (++) when it did not present blinding.
	Quality control	When the diagnosis of malaria was not performed by a health care provider, when analyzes of TNF-α levels were performed by academics in the absence of a supervisor or when it did not cite which professional performed the assessments, a minor problem (+) was considered when one of the previous items was present and a bigger problem (++) when two items were present in the studies.
Completeness	Compliance	It was considered (0) in the studies where the samples remained the same from the beginning to the end, or in a decreasing situation. There was no impairment in the power of the test used, (+) when the sample decrease during the study was able to compromise the. However, there were reasons and adjustments, and (++) was identified when the difference in sample size during the study compromised the power of the test, without justifiable reasons.
	Dropouts	It was considered as (0) when there was no withdrawal during the study, and (++) when there were withdrawals during the study; in cross-sectional studies was assigned Not Applicable (NA).
	Deaths	This item was scored as Not Applicable (NA) due to the type of PECO strategy.
	Missing data	The studies were identified with (0) when there was no loss of data (+) when there was loss without, however, compromising the statistical analysis and (++) when there was loss associated with the impairment of the statistical analysis.
Distorting influences?	Extraneous treatments	(0) was used when there was no influence of external factors, (+) when there were external factors without interfering in the results of the studies and (++) when there were external factors and impairment in the results
	Contamination	This item was scored as Not Applicable (NA) due to the type of PECO strategy.
	Changes over time	It was classified as NA because the studies were performed in a determined period after the diagnosis of *P. falciparum* infection.
	Confounding factors	It is characterized as a confounding factor when the patient of the study presents a clinical picture of previous anemia or when it presents a subclinical inflammatory picture. It was considered (0) when there were no confounding factors, a minor problem (+) when one of the factors was present, and a bigger problem (++) when two factors were present.
	Distortion reduced by analysis	(0) was used when in the studies are cited the adjustment of the variables that present distortions, (+) when the adjustment is cited in the studies, but it does not make clear the criteria used and (++) when the distortions were identified and not adjusted.
Summary questions	Bias: Are the results erroneously biased in a certain direction?	YES or “NO” answers were assigned for each question. If the answer is NO at the three questions, the article is considered reliable, with low risk of bias.
	Confounding: Are there any serious confusing or other distorting influences?	
	Chance: Is it likely that the results occurred by chance?	

Lastly, three questions were answered with YES or NO, aiming to show bias, confounding and probability of chance if NO was answered on all three questions; the study was classified as soundness and with low bias.

### Level of evidence

A summary of the overall certainty of the evidence was presented using “Grading of recommendations, assessment, development and evaluation” (GRADE) tool [27]. Included studies were evaluated according to their design, study quality, consistency, and directness. A comparison between TNF-alpha levels in cerebral malaria and severe malaria was performed in studies evaluating children [28–30] and adults [31–33].

## Results

After search we identified 2338 studies. Most of which were at Scopus (*n* = 1046), followed by Web of Science (*n* = 635), Pubmed (*n* = 248), Google Schoolar (*n* = 237), Cohcrane (*n* = 109) and Open grey (*n* = 01). Alerts did not informed non studies that satisfied the inclusion criterion of this present review.

After the search for duplicated studies, 621 were excluded, remaining 1717 studies. After title and abstract reading, 1682 studies were excluded, and 34 were selected by full reading. The studies selected were: Grau et al. [34], Kwiatkowski et al. [28], Shaffer et al. [35], Molyneux et al. [36], Deloron et al. [37], McGuire et al. [38], Nicolas et al. [39], Baptista et al. [40], Chuncharunee et al. [41], Mordmuller et al. [42], Udomsangpetch et al. [43], Brown et al. [44], Day et al. [45], Maneerat et al. [46], Akanmori et al. [30], May et al. [47], Ubalee et al. [48], Esamai et al. [49], Manish et al. [50], Lyke et al. [51], Armah et al. [52, 53], Cabantous et al. [54], Kinra and Dutta [31], Prakash et al. [32], Hananantachai et al. [55], Tchinda et al. [56], Hassan et al. [57], Mergani et al. [58], Nmorsi et al. [59], Thuma et al. [29], Rovira-Vallbona et al. [60], Pereira et al. [61], Sahu et al. [61].

**Fig. 1:**
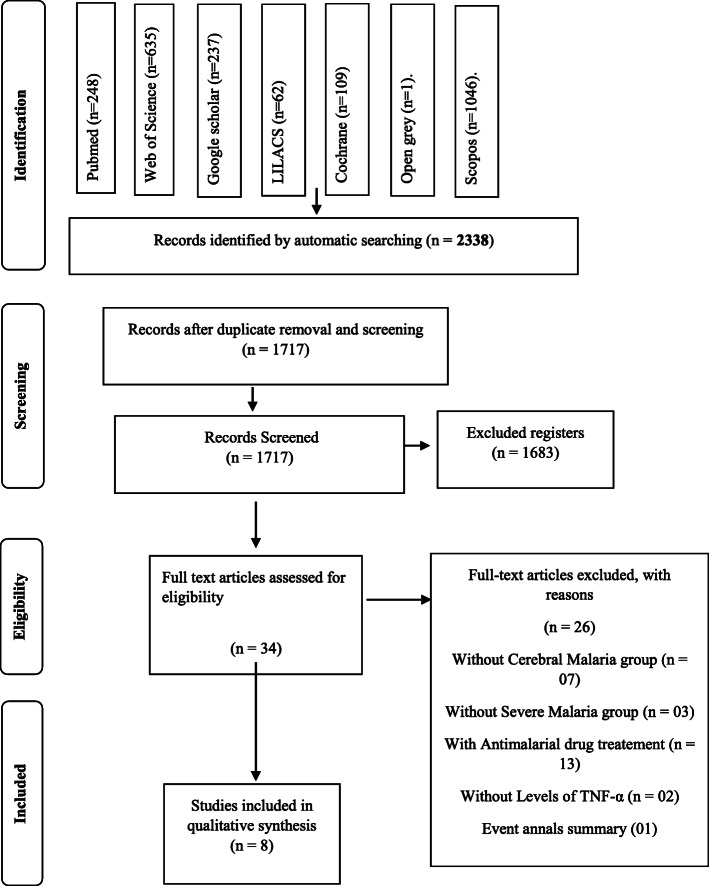
Flowchart diagram of literature search according to PRISMA guidelines

According to the criterion of this present review, seven studies were excluded due to the absence of CM group [36, 42, 45, 51, 57, 59, 61], three due to the absence of reference group without CM [44, 50, 58], 13 studies due to inclusion of antimalarial drug treatment [34, 35, 37, 39–41, 43, 46–48, 56, 59, 60], two due to the absence of TNF-α level quantification [38, 55] and one for being a summary of event annals [52] (Fig. 1).

Only eight studies [28–33, 52–54] **fit in** the criterion of this present review and were **included in** the qualitative analysis. The reference list of each one was checked, and no new study was found. Figure 1 shows the flow chart of each step of the study selection.

### Studies characteristics and risk of bias

After selection of studies, eight [28–33, 52–54] were selected to quality analysis. Among them, Akanmori et al. [30], Armah et al. [52, 53], Cabantous et al. [54], Kwiatkowski et al. [28] and Thuma et al. [29] were performed at Africa, Prakash et al. [32]. Sahu et al. [33] were performed in India, and Kinra and Dutta [31] have not specified where the study was performed. About the population age range, Armah et al. [52, 53], Cabantous et al. [54], Kinra and Dutta [31], Kwiatkowski et al. [28] have not maked it clear in the study, Akanmori et al. [30] have evaluated patients aged 3 to 12 years old, Prakash et al. [32] evaluated patients aged 5 to 75 years old, Sahu et al. [33] patients under 15 years old while Thuma et al. [29] evaluated children under 6 years old. All studies have set up as inclusion criterion of CM group patients in a coma with a score less than two and a severe malaria group with conscious patients, with hepatocytes less than 15% and/or hemoglobin less than 7 g/dl. Besides, seven studies realized TNF-α dosage by Elisa while Armah et al. [52, 53] have performed *a post-Morten* study with TNF-α quantification by immunohistochemistry (Table 3).

**Table 3:** Characteristic of samples and data of included studies

Author, year	Methods of evaluation					Results	Statistical analysis
	Source of sample	Size of sample	Age	Inclusion criterion	Sample type/TNF-α dosage		
**Akinori et al., 2000** [62]	Accra (Hospitalized patients) and Dodowa Community (control group), both in Ghana.	1995: Cerebral Malaria - 41, Severe Malaria – 10; 1997: Cerebral Malaria - 60, Severe Malaria - 24	3 to 12 years	CM: Unrousable coma (Blantyre Coma Score 3/4 3) SM: Haemoglobin < 5 g/dL in fully conscious	Blood/ ELISA Kits	TNF-α levels were significantly higher in CM (147 ± [113–192]) compared to SM (87 ± [54–139]) and UM (77 ± [58–102]).	One-Way and Two-Way Parametric ANOVA following Tukey’s posthoc test. Data were considered significant when *p* < 0,05
**Sample Armah et al., 2005** [52, 53]	Emergency Unit at the Department of Child Health - Korle-Bu Teaching Hospital, Accra, Ghana.	Cerebral Malaria – 10 Severe Malaria - 5	–	CM: Blantyre coma score of 2 SM: Haemoglobin < 5 g/dL in fully conscious	Brain (Post-Morten)/Immunostaining for TNF-alfa	TNF-α was expressed in CM brain sections in the intravascular, perivascular and intraparenchymal pattern, but none of the sections of the other group.	For each of the six antigens studied, the percentage of immunostained cases and the intensity of staining were compared between the five groups of cases and the three regions of the brain.
**Cabantous et al., 2006** [54]	Gabriel Toure and Hospital in Bamako, Mali.	Cerebral Malaria – 58 Severe Malaria - 27	–	CM: Unrousable coma (Blantyre Coma Score 3/4 3) SM: with a thick blood film positive for *P. falciparum*, a packed cell volume of 15%, and no impaired consciousness.	2–5 ml Blood TNF levels by ELISA.	Our analysis also failed to detect significant differences in TNF-α plasma levels between children with CM or SM and control children.	Unmatched groups were compared by using the Mann-Whitney U test (SPSS 10.1 software), with *P values* of < 0.05 being considered significant. The levels of TNF in the plasma of children with CM and SM were low (median 1 pg/ml) and not significantly different.
**Kinra and Dutta, 2013** [31]	This was a multicentric study carried out in three hospitals simultaneously.	Cerebral Malaria – 2 Severe Malaria - 14	–	CM: were comatose and had detectable *P. falciparum* asexual forms in peripheral blood, SM: Subjects with parasite index of more than 1%, blood sugar < 60 mg/dl), severe anaemia (Hb < 7 g/dl)	Plasma sample/ELISA Kits	Compared with the children with uncomplicated malaria, mean plasma TNF-α levels were twice as high in cerebral malaria survivors and ten times as high in the fatal cases.	The nonparametric test (Mann-Whitney rank-sum test) was used for TNF-α analysis. Normally distributed data were analyzed using Student’s t-test (all p values are two-tailed). Pearson’s correlation coefficient was used to evaluate the relationship within normally distributed variables. *P values* < 0.05 were considered significant.
**Kwiatkowski et al., 1990** [28]	Royal Victoria Hospital, Banjul, The Gambia and the Medical Research Council (MRC) Laboratories, Fajara, The Gambia.	Cerebral Malaria – 110; Middle Malaria - 178	–	CM: Coma score of 2 or less, persists for more than 30 min after any convulsions had ceased MM: Febrile illness in a child with asexual, without other satisfactory explanation for the fever and cerebral malária.	Plasma sample/ELISA Kits	In plasma samples collected within 4 h of presentation, the geometric mean TNF-α concentration was higher in malária than in non-malaria patients and increased with the severity of malaria, levels were significantly higher in fatal CM than in non-fatal CM, in non-fatal CM than in MM.	The detection limits of these assays were: TNF- 10 pg/ml; IL-lot 10 pg/ml; and IFN-y 25 pg/ml. For statistical purposes, results below these limits were assigned values of 5 pg/ml, 5 pg/ml, and 12–5 pg/ml, respectively. Data were analyzed with the software package SPSS/PC+ v3.1.
**Prakash et al., 2006** [32]	Gondia District of Maharashtra State, India.	Cerebral Malaria – 26 Severe Malaria - 33	5 to 75 years	CM: were in a comatose state SM: were fully conscious and were able to respond well verbally to the doctors’ questions	Plasma sample/estimated by use of Opti-EIA kits	The levels of IL-1b, IL-10, TNF-α, and TGF-b were observed to be most significantly increased in the CM group, compared with those in the endemic control group or in the other groups of *P. falciparum*-infected patients (for all comparisons, *P* < 10^−6^).	Comparisons between groups were calculated by the Mann-Whitney rank-sum test. The significance criterion was a Bonferroni-corrected *P* value < 0.05. For this purpose, special macros addition to the software IgorPro (version 3.16; WaveMetrics) were used.
**Sahu et al., 2013** [33]	SCB Medical College and Hospital, Cuttack, and Regional Medical Research Centre in Orissa, both in Odisha, India.	Cerebral Malaria – 52 Severe Malaria - 85	< 15 years	CM: was further defined as patients with altered sensorium, GCS (Glasgow Coma Scale) of 610 SM: hemoglobin < 5 g/dl and acute renal failure	Plasma/ ELISA Kits	Significantly elevated levels of TNF-α were noted in all the parasitemic study patients compared to control (12.51 ± 1.779 pg/ml) the highest in MOD (68.57 ± 8.617 pg/ml) followed by CM (52.40 ± 5.285 pg/ml) than the SM	Significance determined by the Kruskal–Wallis test enabled subsequent intergroup comparisons using the Mann–Whitney U test; *P* < 0.05 (2-tailed) was considered significant. The correlations were carried out using the Pearson’s rank test (r) to investigate the relationship between MP counts with clinical as well as biological parameters and TNF-α level. *P* < 0.05(a) was considered significant.
**Thuma et al., 2011** [29]	Pediatric Unit of Yaound’ e Central Hospital, known as The Chantal Biya Foundation, Cameroon.	Cerebral Malaria - 28; Severe Malaria - 72	< 6 years	CM: screening hematocrit level of > 18% and unarousable coma as defined by a Blantyre coma score of < 2 SM: screening hematocrit level of, 15% and the absence of coma	Plasma/Multiplex Immunoassay kits (LINCO Research)	In contrast to those of the children with severe malarial anemia [33.1 (12.3–96.5)], none of the plasma concentrations of the originally selected immune markers differed significantly between children with cerebral malaria [44.7 (35.6–100.4)].	Continuous variables were compared with the Kruskal-Wallis test, and categorical variables were compared with the Fisher exact test. Relationships between 2 continuous variables were assessed with the Spearman correlation coefficient.

*SM* Severe malaria, *CM* Cerebral malaria

All studies were classified as observational. About the representativeness of samples, only Kinra and Dutta [31] showed a major problem of local sampling since it was not clear where the study was performed. Regarding sample size, only Kwiatkowski et al. [28] showed a minor problem due to the sample size greater than 50, while all other studies did not show sample calculation and sample size were up to 50 at least in one group being attributed as a major problem. In the inclusion/exclusion criterion, Kinra and Dutta [31], Kwiatkowski et al. [28] and Prakash et al. [32] showed a minor problem since it was not clear if they included or not a pregnant woman and/or comorbidity and co-infection. The remaining studies, Akanmori et al. [30], Armah et al. [52, 53], Cabantous et al. [54], Sahu et al. [33] and Thuma et al. [29] did not show any problem and none of them have shown problems regarding subjects on the study.

On the question about the acceptance of the control group, only Kinra and Dutta [31] showed a major problem since they did not specify the study region. In the match/randomization question [28–33, 54] showed a minor problem since it was not clear if they matched samples, even though groups were matched. On Armah et al. [52, 53], we identified a major problem since they did not show a proper description and matching of groups.

On the quality of measurements and outcomes, Armah et al. [52, 53] showed a major problem regarding validity since the methods performed to quantified TNF-α does not allow us to make comparison with other studies included in this review. On [28–33, 54], no problem was identified regarding reproducibility and quality control. However, when we evaluated blindness, all studies showed a major problem. Finally, on the compliance topic and distorting influences, none of the studies showed problems.

After evaluation of all topics, we identified that only Armah et al. [52, 53] and Kinra and Dutta [31] had major problems in their studies. On the study performed by Armah et al. [52, 53] we identified major problems (++) regarding sample size, matching/randomization, validity and blindness, while on the study performed by Kinra and Dutta [31] we identified major problems (++) regarding the definition of the source of sample, sample size, and blindness. Minor problems (+) were identified in the inclusion/exclusion criterion and randomization. On the studies performed by Akanmori et al. [30], Cabantous et al. [54], Kwiatkowski et al. [28], Prakash et al. [32], Sahu et al. [33] and Thuma et al. [29] did not show as many problems as Armah (2005a) [52]. They were selected for the evaluation of the association of TNF-α and CM.

Concerning the analysis of the risk of bias, we answered three questions: Are the results erroneously biased in a certain direction? Are there any serious confusing or other distorting influences? Is it likely that the results occurred by chance? To all three questions, the answer was NO, suggesting that none of the selected studies showed a high risk of bias (Table 4).

**Table 4:** Checklist of quality assessment and risk of bias evaluation of included studies according to Fowkes and Fulton [26]

	Checklist	Akinori et al., 2000 [62]	Armah et al., 2005b [53]	Cabantous et al., 2006 [54]	Kinra and Dutta , 2013 [31]	Kwiatkowski et al., 1990 [28]	Prakash et al.,2006 [32]	Sahu et al., 2013 [33]	Thuma et al., 2011 [29]
**Is the study design appropriate to objectives?**	Objective common design								
	Prevalence e Cross-sectional								
	Prognosis Cohort								
	Treatment Controlled trial								
	Cohort, case-control, cross-sectional	0	0	0	0	0	0	0	0
**Study sample representative?**	Source of sample	0	0	0	++	0	0	0	0
	Sampling method	0	0	0	0	0	0	0	0
	Sample size	**++**	**++**	**++**	**++**	+	**++**	**++**	**++**
	Entry criteria/exclusion	0	0	0	+	+	+	0	0
	Non-respondents	0	0	0	0	0	0	0	0
**Control group acceptable?**	Definition of controls	0	0	0	0	0	0	0	0
	Source of controls	0	0	0	++	0	0	0	0
	Matching/randomization	+	++	+	+	+	+	+	+
	Comparable characteristics	0	0	0	0	0	0	0	0
**Quality of measurements and outcomes?**	Validity	0	++	0	0	0	0	0	0
	Reproducibility	0	0	0	0	0	0	0	0
	Blindness	**++**	**++**	**++**	++	**++**	**++**	++	++
	Quality control	0	0	0	0	0	0	0	0
**Completeness**	Compliance	0	0	0	0	0	0	0	0
	Drop outs	0	0	0	0	0	0	0	0
	Deaths	NA	NA	NA	NA	NA	NA	NA	NA
	Missing data	0	0	0	0	0	0	0	0
**Distorting influences?**	Extraneous treatments	0	0	0	0	0	0	0	0
	Contamination	NA	NA	NA	NA	NA	NA	NA	NA
	Changes over time	NA	NA	NA	NA	NA	NA	NA	NA
	Confounding factors	0	0	0	0	0	0	0	0
	Distortion reduced by analysis	0	0	0	0	0	0	0	0
**Summary questions**	Bias:								
	Are the results erroneously biased in a certain direction?	NO	NO	NO	NO	NO	NO	NO	NO
	Confounding:								
	Are there any serious confusing or other distorting influences?	NO	NO	NO	NO	NO	NO	NO	NO
	Chance:								
	Is it likely that the results occurred by chance?	NO	NO	NO	NO	NO	NO	NO	NO

0 = No problem; + = Minor problem; ++ = Major problem; *NA* Not applicable

Among the eight selected studies to the evaluation of TNF-α on study group (with CM) and reference group (without CM), five showed an increase on TNF-α at CM group when compared to non-CM group [28, 30, 32, 33, 52, 53]. On the other hand, three studies did not show a significant difference between groups [29, 31, 54]. Those data suggest that the increase in TNF-α is closely linked with the development of human CM by *Plasmodium falciparum*.

### Analysis of the level of evidence

The levels of TNF-alpha were most increased in cerebral malaria than severe malaria groups for adults and children. The overall level of certainty was classified as low for studies involving adults and children. This result can be associated with the observational nature of the included studies (Table 5).

**Table 5:** Grading of recommendation, assessment, development, and evaluation (GRADE) instrument

Certainty assessment							Impact	Certainty	Importance
Number of studies	Study design	Risk of bias	Inconsistency	Indirectness	Imprecision	Other considerations			
Levels of TNF-a in adult patients									
3	observational studies	not serious	not serious	not serious	not serious	none	In three studies, 80 patients with cerebral malaria and 40 patients with severe malaria were evaluated. The mean TNF alpha level was significantly higher in cerebral malaria than in severe malaria in all evaluated studies. The mean levels of TNF alpha level varied from 52.40 to 53.26 pg/ml in the cerebral malaria group. No information was reported among means of TNF alpha level in severe malaria group.	⨁⨁◯◯ LOW	CRITICAL
Levels of TNF-a in children patients									
4	observational studies	not serious	not serious	not serious	not serious	none	In four studies, 98 patients with cerebral malaria and 101 patients with severe malaria were evaluated. The mean of TNF alpha level, as well as the immunoexpression reported by one study, were significantly higher in cerebral malaria when compared to severe malaria in the evaluated studies. No information was reported among means of TNF alpha level in both groups.	⨁⨁◯◯ LOW	CRITICAL

## Discussion

In this systematic review, we gathered evidence of the association between Cerebral Malaria and TNF-alpha levels. Among the eight elected studies, five reported an increase in TNF-alpha in patients with cerebral malaria compared to patients with malaria who did not develop Cerebral Malaria; another three articles did not show significant differences between the groups. However, even in the face of the association results presented by most of the selected articles, the low level of evidence presented by them does not offer certainty of the results regarding the association of TNF-alpha with cerebral malaria, according to GRADE criteria.

To gather the evidence presented in this article, we opted for a type of bibliographic research recognized today as the top of the evidence pyramid: the systematic review. A systematic review is a type of study that seeks to gather and analyze articles with similar designs, evaluate them methodologically and, if possible, bring them together in statistical analysis. This type of review seeks to synthesize similar primary studies. It is considered the best level of evidence for decision making in questions about therapy and health conduct [63–67]. In this study, it is possible to perform the quality assessment of methodology and risk of bias following a well-established protocol proposed by Fowkes and Fulton [26]. This questionnaire aimed to analyze the experimental design and also to show a summary of overall certainty of evidence by “Classification of recommendations, assessment, development and evaluation” (GRADE) [27]. In this way, this systematic review could bring clarification on the association between TNF-α level and cerebral malaria.

Of the eight eligible studies, only one has not clarified the source of 298 samples. Two were conducted in India, two in Ghana, one in Mali, one in the Republic of 299 Cameroon and one in the Republic of the Gambia (Table 2), (2000), 300 Sahu et al. [33] and Thuma et al. [29] included children under the age of 15,301, Prakash et al. [32] included children and adults (5 to 75 years), while the other 302 studies did not explain the age range of subjects. Malaria is already a serious public health problem. In 2017, WHO estimated 219,000,000 new cases and about 435,000 deaths worldwide, also in 2017, almost half of the world’s population was at imminent risk of malaria. Most cases occur in Sub-Saharan Africa, but there are other regions at risk, such as Southeast Asia, the Eastern Mediterranean, the Western Pacific, and the Americas. In 2017, 90 countries had continuous malaria transmission. According to the last global report issued by WHO, in November 2018, about 219 million malaria cases were registered in 2017, 217 million in 2016, which shows that each year, the numbers increase substantially. PAHO-WHO [68], with a large part caused by *P.falciparum*, the most lethal of the six human malaria parasites [69]. Another important explanation is the presence of the 307 *Anopheles gambiae* mosquito, the most efficient and most difficult to control malaria vector. It is estimated that 1 million people in Africa die each year from malaria 308, with a prevalence of children under 5 years of age [70].

In areas of stable malaria transmission, incredibly Young children are the main group with a major risk of malaria morbidity and mortality. The highest incidence of infection on children is in the first or second year of life when they have not yet acquired proper clinical immunity. This makes the early years particularly dangerous, accounting for 90% of all deaths in Africa. Three reasons for this high mortality rate are the development of cerebral malaria, the frequency of malaria infections that leads to severe anemia and the low weight at birth that is frequently associated with malaria infection during pregnancy [71]. Also, repeated malaria infection makes children more susceptible to another common disease in childhood as diarrhea and respiratory infections [36].

TNF-α is closely associated with the reduced parasitic load. However, it is shown to be associated with the severity of cerebral malaria if production occurs in its later phase, which may suggest an attempt by TNF-α to control infection, and possibly contribute to the reduction of deaths, especially in children under five, who are the most susceptible to this in endemic areas [23, 24, 33].

After we have analyzed the eight selected studies in this systematic review, we identified that the TNF-α level was found to be increased on the cerebral malaria group, suggesting TNF-α level could be closely linked to malaria. In the quality assessment, all studies showed major problems regarding sample size and blindness. The study performed by Kinra and Dutta [31] was the one, which presented the greatest problems among all. In the risk of bias analysis, none of the studies were identified as erroneously skewed in a certain direction, serious influences, other distortions, or that occurred by chance. However, it is important to note that the level of evidence was considered very low to critical.

All studies that have been selected showed their criterion to determine if patients had or not CM. To characterize CM’s development, patients should present a coma score lower than 2 [69]. On the other hand, patients with severe malaria should be conscious of hemoglobin levels lower than 5 g/dl and hematocrit higher than 15%. Those diagnosis criteria are in agreement with the literature [69]. After classifying subjects on CM or reference group, seven studies have performed the TNF-α level dosage at blood by ELISA. Thus, it was possible to suggest that higher TNF-α levels are showed on the CM group when compared to the reference group.

Regarding sample representativeness, one of the major problems identified in all selected studies is related to the absence of sample size calculation fewer than 50. This problem was identified in seven out of eight selected studies. Malaria studies performed in humans are fundamental to have a high sample size to ensure the population’s representativeness [11]. In addition, we identified minor problems in the acceptance of the control group regarding sample matching. That was not clear in all studies, as well as the absence of blindness that was found to be a major problem. Those problems are capable of getting influence on diagnosis, parasite identification and characterization of groups on each study [69]. Despite the identification of high TNF-α level in CM group and the fact that studies did not show a risk of bias or inconsistency, indirection and inaccuracy, the level of evidence was considered low since included studies were classified as observational, which makes it difficult to accurately measure the error rate.

The antigen production induced by CM leads to the activation of the immunologic system, such as monocytes activation that is responsible for pro-inflammatory cytokines such as IL-1, IL-6 and TNF-α [22]. However, the TNF-α role of CM development remains unclear. In this way, this systematic review has been performed a detailed analysis of five out of eight selected studies. It has identified an increasing production of TNF-α on the CM group when compared to the reference group.

Kwiatkowski et al. [28] have shown an increase in TNF-α production in the acute infection phase by *P. falciparum* on children. However, the circulating concentrations are unusually high in the minority of CM patients with CM when compared to the majority that develops fever without severe complication. However, high concentrations of TNF are associated with death even after the correction of parasitemia and glucose concentrations by multivariate analysis. This observation is following the hypothesis that excessive TNF production could contribute to the pathogenesis of malaria.

## Conclusion

We may conclude that the TNF level seems to be associated with cerebral malaria by *P.falciparum.* However, it is still necessary for the development of studies that make use of a greater sample size to have a more reliable and representative analysis. Even malaria is no longer considered a neglected disease; it is still necessary to investigate this association’s temporal course, especially on children under 5 years old.

## Data Availability

All data generated or analyzed during this study are included in this published article.

## Abbreviations

CM: Cerebral malaria
IL: Interleukins
M-CSF: Macrophage colony-stimulating factor
PfEMP1: Parasitized erythrocyte
SM: Severe malaria
TNF-α: Tumor necrosis factor-alpha
WHO: World health organization

## References

[1] Miller LH, Baruch DI, Marsh K, Doumbo OK (2002). The pathogenic basis of malaria. Nature..

[2] White NJ, Pukrittayakamee S, Hien TT, Faiz MA, Mokuolu OA, Dondorp AM (2004). Malaria. Lancet..

[3] Tuteja R (2007). Malaria: an overview. FESB J.

[4] McKenzie FE, Smith DL, O’Meara WP, Riley EM (2008). Strain theory of malaria: the first 50 years. Adv Parasitol.

[5] White NJ (2008). The role of anti-malarial drugs in eliminating malaria. Malar J.

[6] Kantele A, Jokiranta TS (2011). Review of cases with the emerging fifth human malaria parasite, Plasmodium knowlesi. Clin Infect Dis.

[7] Idro R, Jenkins NE, Newton CR (2005). Pathogenesis, clinical features, and neurological outcome of cerebral malaria. Lancet Neurol.

[8] Snow RW, Guerra CA, Noor AM, Myint HY, Hay SI (2005). The global distribution of clinical episodes of Plasmodium falciparum malaria. Nature..

[9] Sullivan D (2010). Uncertainty in mapping malaria epidemiology: implications for control. Epidemiol Rev.

[10] Carvalho LJMC, Moreira AS, DanieL-Ribeiro CT, Martins YC (2014). Vascular dysfunction as a target for adjuvant therapy in cerebral malaria. Mem Inst Oswaldo Cruz.

[11] World Heath Organization. World malaria report 2018. http://www.who.int/mediacentre/factsheets/fs094/en/. Accessed 26 Sept 2018.

[12] de Souza JB, Riley EM (2002). Cerebral malaria: the contribution of studies in animal models to our understanding of immunopathogenesis. Microbes Infect.

[13] Lou J, Lucas R, Grau GE (2001). Pathogenesis of cerebral malaria: recent experimental data and possible applications for humans. Clin Microbiol Rev.

[14] Bondi FS (1992). The incidence and outcome of neurological abnormalities in childhood cerebral malaria: a long-term follow-up of 62 survivors. Trans R Soc Trop Med Hyg.

[15] Schofield L, Grau GE (2005). Immunological processes in malaria pathogenesis. Nat Rev Immunol.

[16] Berendt AR, Ferguson DJ, Newbold CI (1990). Sequestration in Plasmodium falciparum malaria: sticky cells and sticky problems. Parasitol Today.

[17] Hunt NH, Gray GE (2003). Cytokines: accelerators and brakes in the pathogenesis of cerebral malaria. Trends Immunol.

[18] Combes V, Coltel N, Faille D, Wassmer SC, Grau GE (2006). Cerebral malaria: role of microparticles and platelets in alterations of the blood–brain barrier. Int J Parasitol.

[19] Medana IM, Turner GD (2006). Human cerebral malaria and the blood brain barrier. Int J Parasitol.

[20] Martins YC, Smith MJ, Pelajo-Machado M, Werneck GL, Lenzi HL, Daniel-Ribeiro CT, Moura Carvalho LJ (2009). Characterization of cerebral malaria in the outbred Swiss Webster mouse infected by Plasmodium berghei ANKA. Int J Exp Pathol.

[21] van der Heyde HC, Nolan J, Combes V, Gramaglia I, Grau GE (2006). A unified hypothesis for the genesis of cerebral malaria: sequestration, inflammation and hemostasis leading to microcirculatory dysfunction. Trends Parasitol.

[22] Nebl T, De Veer MJ, Schofield L (2005). Stimulation of innate immune responses by malarial glycosylphosphatidylinositol via pattern recognition receptors. Parasitology..

[23] Omer FM, Kurtzhals JA, Riley EM (2000). Maintaining the immunological balance in parasitic infections: a role for TGF-beta?. Parasitol Today.

[24] PAHO-WHO, Information sheet, 2018. https://www.paho.org/bra.../index.phpoption=com_content&view=article&id=5287:malaria-2&Itemid=875. Accessed 23 Apr 2020.

[25] Moher D, Liberati A, Tetzlaff J, Altman DG (2009). PRISMA Group. Preferred reporting items for systematic reviews and meta-analyses: the PRISMA statement. Ann Intern Med.

[26] Fowkes FG, Fulton PM (1991). Critical appraisal of published research: introductory guidelines. BMJ.

[27] Balshem H, Helfand M, Schünemann HJ, Oxman AD, Kunz R, Brozek J (2011). GRADE guidelines: 3. Rating the quality of evidence. J Clin Epidemiol.

[28] Kwiatkowski D, Sambou I, Twumasi P, Greenwood BM, Hill AVS, Manogue KR (1990). TNF concentration in fatal cerebral, non-fatal cerebral, and uncomplicated Plasmodium falciparum malaria. Lancet..

[29] Thuma PE, van Dijk J, Bucala R, Debebe Z, Nekhai S, Kuddo T (2011). Distinct clinical and immunologic profiles in severe malarial anemia and cerebral malaria in Zambia. J Infect Dis.

[30] Akanmori BD, Kurtzhals JAL, Goka BQ, Adabayeri V, Ofori MF, Nkrumah FK (2000). Distinct patterns of cytokine regulation in discrete clinical forms of Plasmodium falciparum malaria. Eur Cytokine Netw.

[31] Kinra P, Dutta V (2013). Serum TNF alpha levels: a prognostic marker for assessment of severity of malaria. Trop Biomed.

[32] Prakash D, Fesel C, Jain R, Cazenave PA, Mishra GC, Pied S (2006). Clusters of cytokines determine malaria severity in Plasmodium falciparum-infected patients from endemic areas of central India. J Infect Dis.

[33] Sahu U, Sahoo PK, Kar SK, Mohapatra BN, Ranjit M (2013). Association of TNF level with production of circulating cellular microparticles during clinical manifestation of human cerebral malaria. Hum Immunol.

[34] Grau GE, Taylor TE, Molyneux ME, Wirima JJ, Vassalli P, Hommel M (1989). Tumor necrosis factor and disease severity in children with falciparum malaria. N Engl J Med.

[35] Shaffer N, Grau GE, Hedberg K, Davachi F, Lyamba B, Hightower AW (1991). Tumor necrosis factor and severe malaria. J Infect Dis.

[36] Molyneux ME, Engelmann H, Taylor TE, Wirima JJ, Aderka D, Wallach D (1993). Circulating plasma receptors for tumour necrosis factor in malawian children with severe falciparum malaria. Cytokine..

[37] Deloron P, Roux Lombard P, Ringwald P, Wallon M, Niyongabo T, Aubry P (1994). Plasma levels of TNF-alpha soluble receptors correlate with outcome in human falciparum malaria. Eur Cytokine Netw.

[38] McGuire W, Hill AVS, Allsopp CEM, Greenwood BM, Kwjatkowski D (1994). Variation in the TNF-α promoter region associated with susceptibility to cerebral malaria. Nature..

[39] Nicolas P, Hovette P, Merouze F, Touze JE, Martet G (1994). Cytokines and malaria. A study of TNF-alpha, IL1-beta, IL6 and IL2R in 28 patients. Bull Soc Pathol Exot.

[40] Baptista JL, Vanham G, Wéry M, Van Marck E (1997). Cytokine levels during mild and cerebral falciparum malaria in children living in a mesoendemic area. Tropical Med Int Health.

[41] Chuncharunee S, Jootar S, Leelasiri A, Archararit N, Prayoonwiwat W, Mongkonsritragoon W (1997). Levels of serum tumor necrosis factor alpha in relation to clinical involvement and treatment among Thai adults with Plasmodium falciparum malaria. J Med Assoc Thail.

[42] Mordmüller BG, Metzger WG, Juillard P, Brinkman BMN, Verweij CL, Grau GE (1997). Tumor necrosis factor in Plasmodium falciparum malaria: high plasma level is associated with fever, but high production capacity is associated with rapid fever clearance. Eur Cytokine Netw.

[43] Udomsangpetch R, Chivapat S, Viriyavejakul P, Riganti M, Wilairatana P, Pongponratn E (1997). Involvement of cytokines in the histopathology of cerebral malaria. Am J Trop Med Hyg.

[44] Brown H, Turner G, Rogerson S, Tembo M, Mwenechanya J, Molyneux M (1999). Cytokine expression in the brain in human cerebral malaria. J Infect Dis.

[45] Day NPJ, Hien TT, Schollaardt T, Loc PP, Van Chuong L, Chau TTH (1999). The prognostic and pathophysiologic role of pro- and antiinflammatory cytokines in severe malaria. J Infect Dis.

[46] Maneerat Y, Pongponratn E, Viriyavejakul P, Punpoowong B, Looareesuwan S, Udomsangpetch R (1999). Cytokines associated with pathology in the brain tissue of fatal malaria. Southeast Asian J Trop Med Public Health.

[47] May J, Lell B, Luty AJF, Meyer CG, Kremsner PG (2000). Plasma interleukin-10: tumor necrosis factor (TNF)-β ratio is associated with TNF promoter variants and predicts malarial complications. J Infect Dis.

[48] Ubalee R, Suzuki F, Kikuchi M, Tasanor O, Wattanagoon Y, Ruangweerayut R (2001). Strong association of a tumor necrosis factor-α promoter allele with cerebral malaria in Myanmar. Tissue Antigens.

[49] Esamai F, Ernerudh J, Janols H, Welin S, Ekerfelt C, Mining S (2003). Cerebral malaria in children: serum and cerebrospinal fluid TNF-α and 22TGF-β levels and their relationship to clinical outcome. J Trop Pediatr.

[50] Manish R, Tripathy R, Das BK (2003). Plasma glucose and tumour necrosis factor-α in adult patients with severe falciparum malaria. Tropical Med Int Health.

[51] Lyke KE, Burges R, Cissoko Y, Sangare L, Dao M, Diarra I (2004). Serum levels of the proinflammatory cytokines interleukin-1 beta (IL-1β), IL-6, IL-8, IL-10, tumor necrosis factor alpha, and IL-12(p70) in Malian children with severe Plasmodium falciparum malaria and matched uncomplicated malaria or healthy controls. Infect Immun.

[52] Armah H, Dodoo AK, Wiredu EK, Stiles JK, Adjei AA, Gyasi RK (2005). High-level cerebellar expression of cytokines and adhesion molecules in fatal, paediatric, cerebral malaria. Ann Trop Med Parasitol.

[53] Armah H, Wiredu EK, Dodoo AK, Adjei AA, Tettey Y, Gyasi R (2005). Cytokines and adhesion molecules expression in the brain in human cerebral malaria. Int J Environ Res Public Health.

[54] Cabantous S, Doumbo O, Ranque S, Poudiougou B, Traore A, Hou X (2006). Alleles 308A and 238A in the tumor necrosis factor alpha gene promoter do not increase the risk of severe malaria in children with Plasmodium falciparum infection in Mali. Infect Immun.

[55] Hananantachai H, Patarapotikul J, Ohashi J, Naka I, Krudsood S, Looareesuwan S (2007). Significant association between TNF-α (TNF) promoter allele (−1031C, −863C, and -857C) and cerebral malaria in Thailand. Tissue Antigens.

[56] Tchinda VHM, Tadem AD, Tako EA, Tene G, Fogako J, Nyonglema P (2007). Severe malaria in Cameroonian children: correlation between plasma levels of three soluble inducible adhesion molecules and TNF-α. Acta Trop.

[57] Hassan DA, Marques C, Santos-Gomes GM, do Rosario VE, Mohamed HS, Elhussein AM (2009). Differential expression of cytokine genes among sickle-cell-trait (HbAS) and normal (HbAA) children infected with Plasmodium falciparum. Ann Trop Med Parasitol.

[58] Mergani A, Khamis AH, Haboor AB, Hashim E, Gumma M, Awadelseed B (2010). Lack of association between - 308 tumor necrosis factor polymorphism and susceptibility to cerebral malaria among central Sudanese children. Int J Genet Mol Biol.

[59] Nmorsi OPG, Isaac C, Ukwandu NCD, Ohaneme BA (2010). Pro-and anti-inflammatory cytokines profiles among Nigerian children infected with Plasmodium falciparum malaria. Asian Pac J Trop Med.

[60] Rovira-Vallbona E, Moncunill G, Bassat Q, Aguilar R, Machevo S, Puyol L (2012). Low antibodies against Plasmodium falciparum and imbalanced pro-inflammatory cytokines are associated with severe malaria in Mozambican children: a case-control study. Malar J.

[61] Pereira MK, Herath NP, Pathirana SL, Phone-Kyaw M, Alles HK, Mendis KN (2013). Association of high plasma TNF-alpha levels and TNF-alpha/IL-10 ratios with TNF2 allele in severe P. falciparum malaria patients in Sri lanka. Pathog Glob Health.

[62] Akinori BD, Kurtzhals JA, Goka BQ, Adabayeri V, Ofori MF, Nkrumah FK, Behr C, Hviid L (2000). Distinct Patterns of Cytokine Regulation in Discrete Clinical Forms of Plasmodium Falciparum Malaria. Eur Cytokine Netw.

[63] Atallah AN, Castro AA (1998). Revisão sistemática da literatura e metanálise. Medicina baseada em evidências: fundamentos da pesquisa clínica.

[64] Mulrow CD (1994). Rationale for systematic reviews. BMJ..

[65] Clarke M, Horton R (2001). Bringing it all together: Lancet-Cochrane collaborate on systematic reviews. Lancet..

[66] Maia LC, Antonio AG (2012). Systematic reviews in dental research. A guideline. J Clin Pediatr Dent.

[67] Harris JD, Quatman CE, Manring MM, Siston RA, Flanigan DC (2014). How to write a systematic review. Am J Sports Med.

[68] PAHO-WHO (2018). World Health Organization/UNICEF.

[69] The Africa malaria report (2019). World Health Organization/UNICEF 200.

[70] World report malaria (2018). World Health Organization/UNICEF.

[71] Steketee RW, Nahlen BL, Parise ME, Menendez C (2001). The burden of malaria in pregnancy in malaria-endemic areas. Am J Trop Med Hyg.

